# Peroxisomal Metabolite and Cofactor Transport in Humans

**DOI:** 10.3389/fcell.2020.613892

**Published:** 2021-01-11

**Authors:** Serhii Chornyi, Lodewijk IJlst, Carlo W. T. van Roermund, Ronald J. A. Wanders, Hans R. Waterham

**Affiliations:** Laboratory Genetic Metabolic Diseases, Amsterdam UMC Location AMC, University of Amsterdam, Amsterdam, Netherlands

**Keywords:** peroxisomes, transporter, metabolism, cofactor, membrane contact sites, carrier, exchanger

## Abstract

Peroxisomes are membrane-bound organelles involved in many metabolic pathways and essential for human health. They harbor a large number of enzymes involved in the different pathways, thus requiring transport of substrates, products and cofactors involved across the peroxisomal membrane. Although much progress has been made in understanding the permeability properties of peroxisomes, there are still important gaps in our knowledge about the peroxisomal transport of metabolites and cofactors. In this review, we discuss the different modes of transport of metabolites and essential cofactors, including CoA, NAD^+^, NADP^+^, FAD, FMN, ATP, heme, pyridoxal phosphate, and thiamine pyrophosphate across the peroxisomal membrane. This transport can be mediated by non-selective pore-forming proteins, selective transport proteins, membrane contact sites between organelles, and co-import of cofactors with proteins. We also discuss modes of transport mediated by shuttle systems described for NAD^+^/NADH and NADP^+^/NADPH. We mainly focus on current knowledge on human peroxisomal metabolite and cofactor transport, but also include knowledge from studies in plants, yeast, fruit fly, zebrafish, and mice, which has been exemplary in understanding peroxisomal transport mechanisms in general.

## Introduction

Peroxisomes are single-membrane bound organelles found in virtually all eukaryotic cells, ranging from unicellular yeasts to plants and mammals, including humans. The organelles are highly dynamic in nature and play an indispensable role in various metabolic pathways. In humans, peroxisomes are responsible for the alpha- and beta-oxidation of specific classes of fatty acids, the synthesis of bile acids and plasmalogens, detoxification of glyoxylate, and H_2_O_2_ metabolism (Wanders et al., 2018). Overall, more than 60 enzymatic activities have been identified in mammalian peroxisomes (Wanders and Waterham, 2006). Some of the responsible enzymes are associated with the outer surface of the peroxisomal membrane, such as fatty acyl-CoA reductase (FAR1), acyl/alkyl DHAP reductase (PexRAP), and some of the acyl-CoA synthetases, but most peroxisomal enzymes reside in the peroxisomal matrix. The metabolic reactions they catalyze require the import and export of substrates, products, and cofactors across the peroxisomal membrane. Transport of bulky molecules is achieved by specialized transporter proteins located in the peroxisomal membrane. However, for the transport of small molecules two types of transport proteins appear to function simultaneously in the peroxisomal membrane: size-selective pore-forming proteins and carrier proteins. Ever since it was first postulated 30 years ago (van Veldhoven et al., 1987), passive diffusion through pore-forming proteins has remained hotly debated (see “Pore-Forming Proteins”). However, very few carrier proteins have been identified in the peroxisomal membrane, and these proteins alone cannot account for the transport of the large variety of molecules involved in enzymatic reactions within peroxisomes (see “SLC Family of Mitochondrial Solute Transporters”).

Proteomic analysis of mammalian peroxisomes together with sequence-based prediction tools and studies of the individual proteins has led to the identification of some peroxisomal metabolite transporters. Based on their properties and/or sequence similarity, these membrane proteins can be categorized into four groups of transporters, which will be discussed separately. The first group includes membrane proteins that show an ability to form pore-like channels and facilitate the passage of small metabolites (Pex11beta, PXMP2 (PMP22), BAK). The second group includes active transporters that use ATP-hydrolysis as an energy source [ABCD1 (ALDP), ABCD2 (ALDRP), and ABCD3 (PMP70)]. The third group includes proteins that, based on sequence similarity, belong to the different families of mitochondrial solute transporters [SLC25A17 (PMP34), MCT1 (SLC16A1), MCT2 (SLC16A7)]. Finally, the fourth group includes several uncharacterized membrane proteins that may play a role in peroxisomal transport [PXMP4 (PMP24), TMEM135 (PMP52)].

The main function of the metabolite transporters in peroxisomes is direct transport of the metabolites across the membrane. In addition, in analogy to the situation in mitochondria, the existence of specific peroxisomal shuttle systems in mammals (Gee et al., 1974), yeasts (van Roermund et al., 1995), and plants (Pracharoenwattana et al., 2007, 2010) has been reported or predicted, which are responsible for small metabolites-mediated reoxidation of NADH and reduction of NADP^+^. In contrast to yeasts and plants, for humans the existence of such shuttle systems still needs to be demonstrated convincingly.

In this review, we discuss the currently known peroxisomal metabolite transporters with focus on the transport of cofactors and describe the possible involvement of shuttle systems. We will focus mainly on such proteins in humans and partly in plants, yeast, fruit fly, zebrafish, and mice. For previous reviews on peroxisomal metabolite transport we refer to Antonenkov and Hiltunen (2006, 2012), Visser et al. (2007b), Plett et al. (2020), and for a specialized review on peroxisomal metabolite transporter proteins in plants, we refer to an excellent review by (Charton et al., 2019).

## Pore-Forming Proteins

The most widely accepted mechanism of transfer of small metabolites across the peroxisomal membrane is via size-selective pore-forming channels. This mechanism of transfer is involved in both import and export of metabolites. However, it is not clear if all low-molecular-weight metabolites cross the peroxisomal membrane through non-selective pores, or that for some of these selective transporters are required.

The first indication for pore-forming proteins in the peroxisomal membrane was provided by de Duve and Baudhuin (1966) who found that purified peroxisomes are permeable to sucrose and pose much higher permeability to H_2_O_2_ than other membranes. Later, van Veldhoven et al. (1987) determined in more detail the permeability properties of purified peroxisomes and liposomes with reconstituted peroxisomal membranes. They observed rapid uptake of a wide range of low-molecular-weight solutes and proposed that peroxisomes contain pore-forming proteins.

The concept that peroxisomes are permeable to low molecular weight metabolites was largely based on *in vitro* experiments with purified peroxisomes. It is known, however, that peroxisomes lose their structural integrity upon cell homogenization and subsequent purification procedures. To overcome this potential problem, Verleur and Wanders (1993) designed a semi-intact cell system in which the cellular membrane of rat hepatocytes was permeabilized with digitonin, while the integrity of peroxisomes remained intact. In these studies, different peroxisomal enzymes including urate oxidase, D-amino acid oxidase, and L-hydroxy-acid oxidase did not show structure-linked latency, which suggested free, unrestricted transfer of urate, D-alanine, and glycolate across the peroxisomal membrane. In contrast, the enzyme dihydroxyacetone phosphate acyltransferase, which generates acyl-DHAP from palmitoyl-CoA and DHAP, did show structure-linked latency when studied in digitonin-permeabilized human skin fibroblasts. The subsequent finding that the addition of (Mg++)ATP resolved the structure-linked latency provided the first indication of ATP-driven transport of acyl-CoAs across the peroxisomal membrane (Wolvetang et al., 1990, 1991).

Perhaps the most compelling evidence in favor of the functional presence of pore-forming proteins *in vivo* was provided by DeLoache et al. (2016). In this study an ingenious approach was used in which β-glucosidase was targeted to yeast peroxisomes by means of a specifically designed enhanced peroxisomal targeting signal type 1 (PTS1). To allow uptake of the substrates of β-glucosidase by the yeast cells, the β-glucosidase-PTS1 was co-expressed with the appropriate transporter targeted to the cell membrane. The substrates for β-glucosidase tested in this cell model were all labeled with the dye 5-bromo-4-chloro-indoxyl (BCI) that becomes fluorescent after hydrolysis by β-glucosidase. It was found that BCI-labeled glucoside (409 Da) and -cellobioside (571 Da) were hydrolyzed by the peroxisomal β-glucosidase and thus able to enter the peroxisomal matrix, whereas the larger molecule BCI-labeled cellotrioside (733 Da) showed restricted permeability. Interestingly, the peroxisomal membrane was also found permeable to L-tryptophan (204 Da) and 2-imino-3-(indol-3-yl)propanoic acid (202 Da) but not to 2,5-diiminio-3,4-bis(indol-3-yl)hexanedioate (402 Da). As the size of the latter molecule is similar to that of BCI-labeled glucoside, these findings suggest that the permeability of the peroxisomal membrane for molecules may not only depend on size, but also on other physical properties, such as shape or charge of the molecules.

Over the years, a number of experiments have shown restricted permeation of the peroxisomal membrane to H_2_O_2_ (Antunes and Cadenas, 2000; Branco et al., 2004; Fransen and Lismont, 2018). Furthermore, the existence of a proton gradient (Dansen et al., 2000; Lasorsa et al., 2004; van Roermund et al., 2004; Godinho and Schrader, 2017), and a Ca^2+^gradient (Lasorsa et al., 2008) had been reported for peroxisomes. It should be noted, however, that such proton and Ca^2+^ gradients could also be the consequence of a difference in the number of protons and Ca^2+^ ions coupled to bulky molecules (e.g., lipids, proteins) localized inside and outside the peroxisomes and thus does not necessarily require the peroxisomal membrane to be impermeable to these ions (Rokka et al., 2009).

**PXMP2** (PMP22) was the first reported peroxisomal protein with pore-forming activity. The diameter of the PXMP2 pore was estimated to be 1.4 nm with selectivity to solutes with molecular size below 600 Da (Figure 2). In an attempt to elucidate its physiological role, PXMP2 was deleted in mice. The Pxmp2 knock-out mice showed a virtually normal phenotype, which was explained by the authors as a consequence of functional redundancy due to the presence of different pore-forming proteins in peroxisomes which compensate for the loss of PXMP2 (Rokka et al., 2009; Vapola et al., 2014). However, the PXMP2 deficiency in mice was associated with severe problems in mammary glands development and decreased levels of myristic acid and some diacylglycerols and phospholipids with polyunsaturated fatty acids in the mammary fat pad. This suggests that the redundancy brought forward by Rokka et al. (2009) may differ per cell- and tissue type (Vapola et al., 2014).

Another human peroxisomal protein that may form a size-selective channel is **Pex11**. In yeast mutants with a deletion of Pex11, the beta-oxidation of fatty acids is affected in whole cells, but in cell lysates normal beta-oxidation was found (van Roermund et al., 2000). This may be explained by an involvement of Pex11 in the transport of some of the metabolites across the peroxisomal membrane. Mindthoff et al. (2016) reported that Pex11 from yeast actually forms a membrane channel with the ability to transport non-selectively metabolites with a molecular weight below 400 Da.

Human peroxisomes contain three different Pex11 isoforms: Pex11α, Pex11β, and Pex11γ (Tanaka et al., 2003; Figure 2). Of these, Pex11β is best studied. The protein is widely expressed and plays an essential role in peroxisome proliferation. Loss of Pex11β results in fewer peroxisomes which are bigger in size in cells, whereas clinically the patients show resemblance to patients with a Zellweger spectrum disorder (Ebberink et al., 2012). Pex11γ was found to have a similar function as Pex11β and also appears to be involved in the elongation of peroxisomes (Koch et al., 2010; Koch and Brocard, 2012). Yet, Pex11γ cannot functionally complement Pex11β deficiency (Ebberink et al., 2012) and so far no patients with a deficiency of PEX11γ have been found. Pex11α shows tissue-specific expression, mainly in adipose tissue, liver, kidney, heart, gastrocnemius, and brain (Chen et al., 2018), and its function is not yet well described. Similar to other Pex11 isoforms, Pex11α appears to be involved in peroxisomal proliferation as overexpression of Pex11α induces the formation of smaller peroxisomes (Koch et al., 2010). Interestingly, Pex11α knock-out mice show an accumulation of very long- and long-chain fatty acids and develop obesity due to dyslipidemia (Chen et al., 2018). While the observed changes in metabolism of Pex11-deficient cell lines may be caused by dysfunction of peroxisomal fission, it may also be related to the involvement of Pex11 in the transport of metabolites. The high level of protein sequence similarity between yeast and human makes the human PEX11 proteins candidates for a non-selective peroxisomal channel.

To study the possibility that PXMP2 and Pex11β are involved in H_2_O_2_ export, the two encoding genes have been deleted in the human cell line HEK293 in which the enzyme D-amino acid oxidase is overexpressed to induce intraperoxisomal H_2_O_2_ levels. Intraperoxisomal H_2_O_2_ was measured *in vivo* using a fluorescent H_2_O_2_ biosensor targeted to peroxisomes. Single and double deletions of both PXMP2 and Pex11β did not lead to any change in the concentration of intraperoxisomal H_2_O_2_ (Lismont et al., 2019a, b), which most probably excludes a role of these two proteins in H_2_O_2_ export. These findings also indicate that PXMP2 and Pex11β are not required for the import of D-alanine, the substrate of D-amino acid oxidase.

Hosoi et al. (2017) recently reported that the mitochondrial protein **BAK** may regulate peroxisomal permeability. BAK is a proapoptotic protein that belongs to the Bcl-2 family and is able to oligomerize to form a membrane pore. Mitochondrial localization of BAK is regulated by the mitochondrial membrane protein VDAC2 that stabilizes the mitochondrial targeting signal of BAK (Roy et al., 2009; Naghdi et al., 2015). Surprisingly, a deletion of VDAC2 in CHO cell lines resulted in a predominantly peroxisomal localization of BAK. Subsequently, it was found that BAK was also localized to peroxisomes in wild-type CHO and HeLa cells (Figure 2). When overexpressed BAK is targeted to peroxisomes by fusion to the peroxisomal membrane protein Pex26, it induces the release of peroxisomal matrix proteins, such as catalase, into the cytosol (Hosoi et al., 2017).

Peroxisomes possess a unique machinery for the import of peroxisomal matrix proteins, which involves the generation of transiently formed membrane pores that allow import of fully folded proteins or even protein complexes. Two different types of pores can be formed in order to import proteins targeted by PTS1 and PTS2 signals, respectively, with an estimated PTS2 pore size in *S. cerevisiae* of ∼4.7 nm (Montilla-Martinez et al., 2015). Remarkably, the transient and possibly selective nature of this pore allows translocation of proteins without apparent major leakage of metabolites *in vivo*.

## Peroxisomal Solute Import

### Fatty Acid Import and the Role of the ABCD Transporters

**FIGURE 1:**
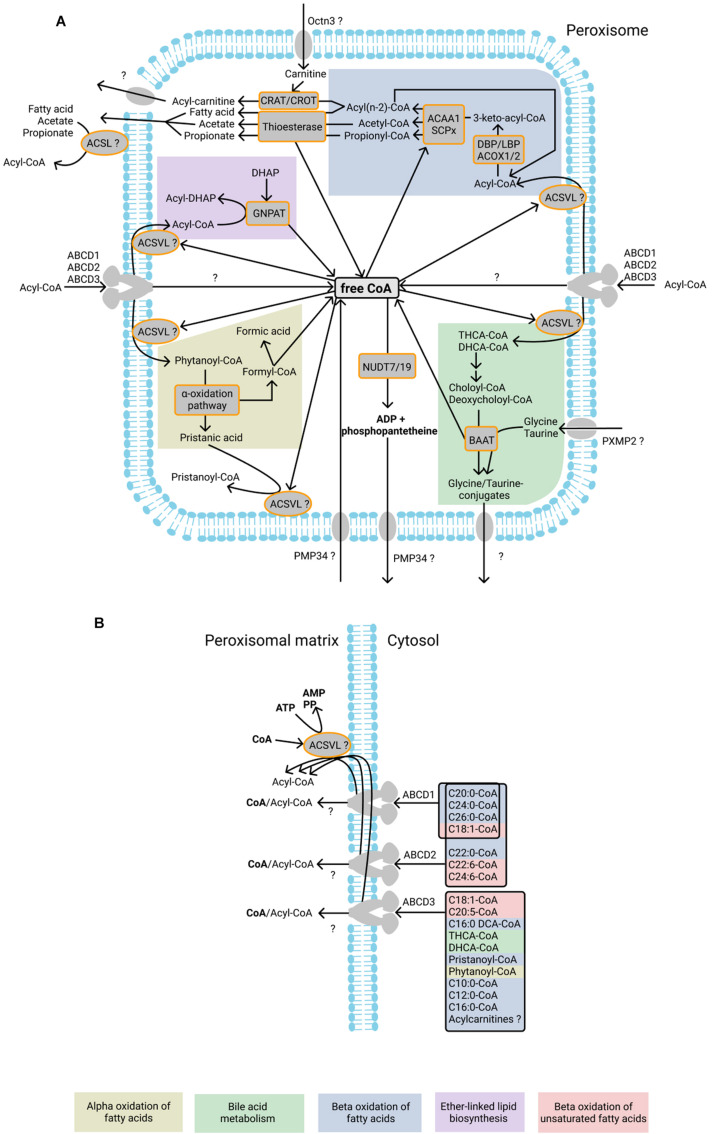
Currently known CoA-dependent enzymatic reactions and transporters in human peroxisomes. **(A)** Fatty acids undergo beta-oxidation after import as acyl-CoA esters into peroxisomes. During beta-oxidation, acyl-CoAs are shortened to acyl(n-2)-CoA and acetyl-CoA molecules are produced. Peroxisomal beta-oxidation is mediated by the enzymes acyl-CoA oxidase 1, 2, and 3 (ACOX1, ACOX2, and ACOX3), L- and D-bifunctional protein (LBP and DBP), acetyl-CoA acyltransferase 1 (ACAA1), and sterol carrier protein x-related thiolase (SCPx). One CoA molecule is required for each circle of beta-oxidation. Acyl-CoA molecules produced during beta-oxidation may be hydrolyzed by thioesterases into fatty acids or acetate or converted to carnitine esters by CRAT and CROT, thereby producing free CoA. Free fatty acids and acetate can probably exit peroxisome directly after which ACSL, located on the cytosolic side of peroxisomal membrane, may be involved in the reactivation of the fatty acid and acetate. It is unclear which transporter is responsible for the export of acyl-carnitines. The peroxisomal enzyme GNPAT uses acyl-CoA esters for the acylation of DHAP during ether-linked lipid biosynthesis. During this reaction free CoA is released. Phytanoyl-CoA undergoes alpha-oxidation inside peroxisomes, resulting in the formation of formyl-CoA, which spontaneously splits into formic acid and CoA. Another product of alpha-oxidation – pristanic acid - is reactivated into pristanoyl-CoA by peroxisomal ACSVL. The bile acids THCA-CoA and DHCA-CoA are subjected to one cycle of beta-oxidation in peroxisomes during which choloyl-CoA and deoxycholoyl-CoA are produced and subsequently converted to glycine or taurine conjugates by BAAT. It is unknown how the conjugates are exported from the peroxisomes. It has been suggested that PMP34 imports free CoA into peroxisomes. The CoA diphosphohydrolases NUDT19 and NUDT7 degrade CoA into 3′,5′-ADP, and 4′-phosphopantetheine, which are subsequently exported from peroxisomes, possibly by PMP34. Proteins of the ABCD subfamily import acyl-CoA molecules into peroxisomes. During the import, the ester bond of acyl-CoA is hydrolyzed, and fatty acids subsequently undergo re-esterification by the intraperoxisomal ACSVL proteins. It is unknown whether the CoA molecule from acyl-CoA is also imported into the peroxisomal matrix after hydrolysis. **(B)** ABCD1, ABCD2, and ABCD3 transporters have different substrate affinities, as shown on the right. After the import of fatty acids, they are esterified by the ACSVL proteins in a CoA- and ATP-dependent reaction. ABCD proteins were shown to hydrolyze the ester bond in the CoA esters during import, although it has also been suggested that ABCD transporters import acyl-CoA molecules without hydrolysis of the ester bond (see text for more information). Enzymatic reactions or molecules belonging to the same metabolic pathway are marked with background color and listed at the bottom.

In humans, beta-oxidation of fatty acids occurs inside mitochondria and peroxisomes. Peroxisomes in particular handle fatty acids that cannot be degraded in mitochondria (1) very-long-chain fatty acids, which are fatty acids with carbon chains of at least 22 atoms long; (2) branched-chain fatty acids like pristanic acid (2,6,10,14-tetramethylpentadecanoic acid); (3) the bile acid intermediates di- and trihydroxycholestanoic acid and (4) long-chain dicarboxylic acids (see also review by Wanders et al., 2018). The peroxisomal import of fatty acids is mediated by three peroxisomal members of the ABC (ATP-binding cassette) superfamily: ABCD1 (also known as ALDP), ABCD2 (also known as ALDR), and ABCD3 (also known as PMP70) (Figure 1A).

ABC transporters form one of the largest superfamilies of membrane transporters. Of the four members of the ABCD sub-family encoded in the human genome, ABCD1-3 are localized exclusively in peroxisomes, while ABCD4 is localized in the lysosomal membrane. ABCD1-3 are half ABC transporters that require homo- (van Roermund et al., 2011, 2014) or heterodimerization (Hillebrand et al., 2007) to form a functional full ABC transporter that contains a dimeric nucleotide-binding domain (NBD) and a transmembrane domain, that consists of two six-transmembrane helices. The general mechanism of transport mediated by ABC transporters involves binding and hydrolysis of ATP that is coupled to conformational changes in the transmembrane domain (TMD), which cause the formation of outward- and inward-facing conformations that mediates the transport of the substrates (Hollenstein et al., 2007). Transporters with greater substrate affinity in the inward-facing conformation, including ABCD transporters, are classified as ABC exporters.

### ABCD1 and ACBD2

ABCD1 and ABCD2 share significant sequence similarity and have been shown by functional complementation experiments to exhibit overlapping substrate specificities (Morita and Imanaka, 2012).

The *ABCD1* and *ABCD2* genes have different expression patterns that also vary during brain development. In the adult, *ABCD1* is expressed mainly in the adrenal gland, heart, intestine, kidney, liver, lung, placenta, and testis, while *ABCD2* is expressed in the adrenal gland, brain, heart, liver, lung, and skeletal muscle (reviewed by Kemp et al., 2011). Interestingly, the expression of *ABCD1* in the brain is highest at birth and decreases over time, while ABCD2 expression increases after birth (Berger et al., 1999). Also, expression profiles differ between cell types; *ABCD1* is expressed in astrocytes, microglial cells, and Schwann cells, but not in neurons, while *ABCD2* is expressed in neurons, astrocytes, and microglia (Fouquet et al., 1997; Troffer-Charlier et al., 1998; Kemp et al., 2011).

Mutations in the *ABCD1* gene result in the human neurodegenerative disorder X-linked adrenoleukodystrophy (X-ALD); so far no disease has been linked to mutations in the ABCD2 gene. This may relate to metabolic aberrations occurring in cell types in which there is no co-expression of *ABCD2* with *ABCD1*. X-ALD is the most frequently occurring peroxisomal disorder and characterized by the accumulation of very long-chain fatty acids (VLCFAs) in the brain, adrenal glands, and plasma (Kemp et al., 2012). Although the accumulation of VLCFAs in X-ALD patients pointed to a role of ABCD1 in transporting VLCFAs across the peroxisomal membrane, the actual transport mechanism has long remained unclear, mainly because the hydrophobic nature of VLCFAs makes classical *in vitro* transport experiments with liposomes difficult. However, the transport of fatty acids by human ABCD1 and ABCD2 could be resolved *in vivo* by means of functional expression in the yeast *Saccharomyces cerevisiae* (van Roermund et al., 2008). In contrast to human cells which can beta-oxidize fatty acids both in mitochondria as well as in peroxisomes, beta-oxidation of fatty acids in *S. cerevisiae* is exclusively peroxisomal. Pxa1p and Pxa2p are yeast orthologs of the human ABCD proteins and are essential for the peroxisomal import of fatty acids as their corresponding acyl-CoA esters. After deletion of the two genes encoding the yeast Pxa1 and Pxa2 proteins, the resulting *pxa1*Δ*/pxa2*Δ cells are no longer able to grow on fatty acids, including oleate, as sole carbon source, and are deficient in fatty acid beta-oxidation. Expression of human ABCD1 or ABCD2 in the *pxa1*Δ*/pxa2*Δ cells was found to rescue the beta-oxidation of a number of fatty acids. These studies showed that ABCD1 and ABCD2 have overlapping specificities but different preferences: ABCD1 expression rescued beta-oxidation of saturated very-long chain fatty acids C24:0 and C26:0 best, whereas ABCD2 expression was best in rescuing the beta-oxidation of C22:0 and poly unsaturated fatty acids C22:6 and C24:6 (van Roermund et al., 2008, 2011, 2014). This is in line with the finding that ABCD1-deficient fibroblasts predominantly accumulate C24:0 and C26:0 (Ofman et al., 2010). The substrate preference of ABCD2 for unsaturated fatty acids is also in agreement with the reported decreased levels of docosahexaenoic acid C22:6-omega-3 in Abcd2-deficient mice (Fourcade et al., 2009; Figure 1B).

An additional indication for functional overlap between ABCD1 and ABCD2 comes from the finding of a more severe phenotype in mice with combined ABCD1 and ABCD2 deficiency in comparison to single ABCD1 or ABCD2 deficiency (Pujol et al., 2004). In line with the somewhat overlapping, yet distinct functions of ABCD1 and ABCD2, a double knock out of the *ABCD1* and *ABCD2* genes in microglial cells (BV-2 cell line) was found to lead to the accumulation of both VLCFAs and PUFAs, while only minor changes were found in cells with a single knock out of *ABCD1* or *ABCD2* in this study (Raas et al., 2019). Also, overexpression of ABCD2 in X-ALD fibroblasts restored beta-oxidation of VLCFA (Netik et al., 1999).

Partial functional overlap between ABCD1 and ABCD2 was also shown in experiments with overexpression of ABCD2-GFP under control of a doxycycline-inducible promoter in the H4IIEC3 rat hepatoma cell line. The expression level of ABCD2 inversely correlated with the abundance of not only C24:1 and C26:1 but also C24:0 and C26:0 (Genin et al., 2011).

Upon incubation with oleate, *pxa1*Δ*/pxa2*Δ yeast cells show an accumulation of oleoyl-CoA (C18:1-CoA), while the levels of the first intermediate of beta-oxidation, i.e., 2-enoyl-CoA (C18:2-CoA) generated by acyl-CoA oxidase, were lowered. This strongly suggests that fatty acids are imported by Pxa1p/Pxa2p in the form of CoA-esters. The introduction of human ABCD1 into this yeast strain resulted in a restoration of the C18:2-CoA/C18:1-CoA ratio, which indicates that ABCD1 is also transporting acyl-CoA esters (van Roermund et al., 2008; Figure 1B).

### ABCD3

The role of ABCD3 in the transport of fatty acids was suggested after the overexpression of human ABCD3 in Chinese hamster ovary (CHO) cells. Overexpression of wild-type ABCD3 led to a two-fold increase of beta-oxidation of palmitic acid while expression of ABCD3 containing a mutation in the Walker A motif, essential for ATP-binding, reduced beta-oxidation (Imanaka et al., 1999).

Expression in yeast showed that the substrate specificity of ABCD3 partially overlaps with that of ABCD1 and ABCD2 but differs in preference (van Roermund et al., 2014). ABCD3 has substrate specificity towards the CoA-esters of long-chain saturated and long-chain unsaturated (C16:0, C18:1, C18:2, C20:5, C22:6), long-branched chain (pristanic acid), and long-chain dicarboxylic fatty acids (C16:0 DCA) (van Roermund et al., 2014; Figure 1B). Inhibition of ABCD3 by means of a specific antibody substantially decreased beta-oxidation of C26:0 in isolated peroxisomes but not in homogenates from X-ALD fibroblasts, which suggests that ABCD3 accounts for the residual beta-oxidation activity (about 30%) measured in these fibroblasts (Wiesinger et al., 2013). Transport activity by ABCD3, as well as by the other ABCD proteins, is dependent on intra-peroxisomal acyl-CoA synthetase activity, which implies that ABCD3-mediated transport involves a re-esterification step. This aspect was specifically addressed by van Roermund et al. (2012) who provided direct experimental evidence in favor of this postulate as discussed below under “ATP Transport” (Figure 1B).

Transport of long-branched chain fatty acids across the peroxisomal membrane is mediated preferentially by ABCD3. This was confirmed by findings in cells of a patient with a genetically determined deficiency of ABCD3, which revealed reduced beta-oxidation of the long-branched chain fatty acid pristanic acid, whereas C26:0 beta-oxidation was normal in these cells. Furthermore, supplementation of phytol (precursor of phytanic acid) to the diet of *Abcd3*(-/-) mice led to much higher accumulation of phytanic acid and pristanic acid than in wild type mice (Ferdinandusse et al., 2015).

Measurement of the peroxisomal biomarkers in plasma from the ABCD3-deficient patient revealed the accumulation of di-and trihydroxycholestanoic acid in line with the presumed role of ABCD3 in the transmembrane transport of these bile acid intermediates. Similar observations were made in the Abcd3-/- mice which accumulated C27-bile acids in the liver, bile, and intestine, whereas the concentration of the different C24 bile acids was reduced (Ferdinandusse et al., 2015).

Under normal conditions, peroxisomal beta-oxidation is the exclusive route for the oxidation of VLCFAs, long-branched chain fatty acids and dicarboxylic acids, and mitochondrial fatty acid beta-oxidation the major route for oxidation of short-, medium-, and long-chain fatty acids in humans. However, when mitochondrial beta-oxidation is compromised, as in the mitochondrial beta-oxidation deficiencies, peroxisomal fatty acid oxidation may serve as an alternative route for the oxidation of short-, medium-, and long-chain fatty acids (Violante et al., 2013, 2018). This was shown elegantly by combining a defective mitochondrial fatty acid oxidation, generated by introducing a CPT1A deletion or by adding a CPT1 inhibitor, with a defective peroxisomal beta-oxidation, generated by disrupting the gene coding for *PEX13*, essential for peroxisome biogenesis. While the CPT1A-depleted cells still oxidized medium- and long-chain fatty acids, this oxidation was completely deficient when *PEX13* was depleted as well. In HEK293 cell lines with defects in mitochondrial fatty acid oxidation, ABCD3 was shown to be crucial for the transport of mitochondrial fatty acids substrates (C10:0, C12:0, C16:0) into peroxisomes. Remarkably, these experiments indicated that ABCD3 can transport these fatty acids not only in the form of CoA-esters but also as acylcarnitine esters (Violante et al., 2018; Figure 1B).

### SLC Family of Mitochondrial Solute Transporters

The SLC (SoLute Carrier) family is the second-largest family of membrane proteins in the human genome consisting of over 450 members. Members of the family include passive transporters, symporters, and exchangers but not primary active transporters, ion channels, or aquaporins (Höglund et al., 2011). So far, the location of five members of three sub-families of SLC proteins (SLC16, SLC25, and SLC27) have been reported in the human peroxisomal membrane, although the evidence is not always that conclusive.

### SLC16 Transporters

**FIGURE 2:**
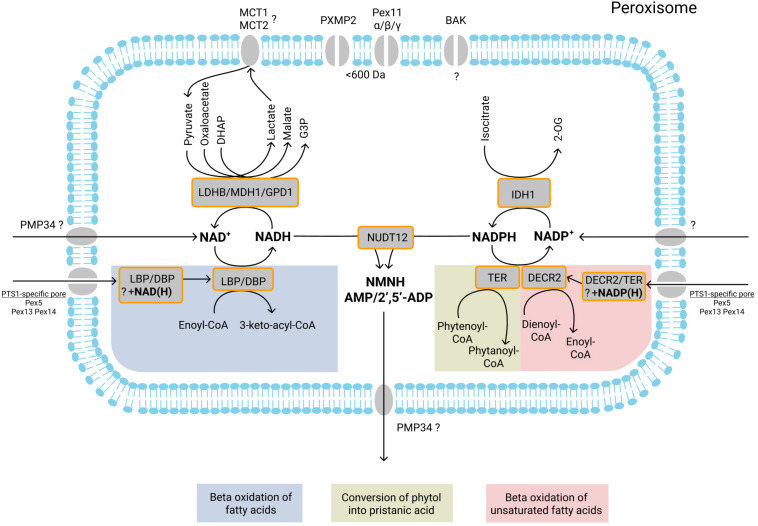
Currently known NAD^+^/NADH- and NADP^+^/NADPH-dependent peroxisomal enzymatic reactions, transport of NAD^+^/NADH, NADP^+^/NADPH, NMNH^+^, AMP, and 2′,5′-ADP, and role of shuttle systems. NAD^+^/NADH and NADP^+^/NADPH may be imported into peroxisomes by specific transporters or co-imported with fully folded NAD(H)/NADP(H)-dependent enzymes. During peroxisomal beta-oxidation, D- and L-bifunctional proteins (DBP and LBP) catalyze the second (hydration) and third (dehydrogenation) step of beta oxidation. During the dehydrogenation, NAD^+^ is reduced to NADH. Subsequent reoxidation of NADH may be mediated by the three different dehydrogenases located inside peroxisomes: LDHB, which converts pyruvate into lactate; MDH1, which converts oxaloacetate into malate; GPD1, which converts DHAP into G3P. During beta-oxidation, the double bound(s) of mono-/polyunsaturated fatty acids are reduced by the peroxisomal enzyme 2,4- dienoyl-CoA reductase (DECR2). During the reduction, NADPH is oxidized to NADP^+^. NADPH is also oxidized by TER during convertion of phytenoyl-CoA into phytanoyl-CoA. NADP^+^ is reduced back to NADPH by IDH1, which converts isocitrate into 2-OG. The import and export of small metabolites [pyruvate, lactate, oxaloacetate, malate, DHAP, G3P, isocitrate, and 2-oxoglutarate (2-OG)] is probably mediated by the peroxisomal pore-forming proteins (PXMP2, Pex11α, Pex11β, and Pex11γ) that allow passage of molecules with a size below 600 Da. The role of another pore-forming protein BAK in the permeability of the peroxisomal membrane remains unclear. Monocarboxylate transporters MCT1 and MCT2 may be involved in the transport of lactate and pyruvate. The pyrophosphatase NUDT12 mediates NADH degradation (to NMNH and AMP) and NADPH degradation (to NMNH and 2’,5′-ADP). The products of NADH/NADPH degradation are subsequently exported from peroxisomes, probably by PMP34. Enzymatic reactions or molecules belonging to the same metabolic pathway are marked with background color and listed at the bottom.

The SL16 family includes the monocarboxylate transporters (MCTs), which are secondary active transporters that display a broad range of specificity to small metabolites and, for their function, depend on proton-gradients. A few studies have reported the presence of MCT1 and MCT2 in the peroxisomal membrane (Figure 2).

MCT1 and MCT2 share 59 percent of amino acid identity and have overlapping functions (Felmlee et al., 2020). MCT1 (SLC16A1) displays specificity to a number of substrates: lactate, pyruvate, acetoacetate, 2-oxoisohexanoate, 2-oxoisovalerate, and butyrate (Bröer et al., 1998) (Borthakur et al., 2006). The protein is expressed in most tissues (Morris and Felmlee, 2008; Uhlén et al., 2015) and mainly found in the plasma membrane (Kirk et al., 2000) and the nucleus (Valença et al., 2015; Thul et al., 2017). This transporter is classified as a H^+^ exchanger and is also involved in pH homeostasis control (Chatel et al., 2017). Several patients with MCT1 deficiency have been reported, who display predominantly ketoacidosis with increased excretion of 3-hydroxybutyrate and acetoacetate (van Hasselt et al., 2014). Of note, the patients did not show metabolic aberrations that can be attributed to metabolic dysfunctioning of peroxisomes.

MCT2 (SLC16A7) is a high-affinity pyruvate (Lin et al., 1998) and lactate (Bröer et al., 1999) transporter, also localized mainly in the plasma membrane. MCT2 from rat also shows an ability to transport β-hydroxybutyrate, acetoacetate, 2-oxoisovalerate, and 2-oxoisohexanoate (Bröer et al., 1999). Expression of MCT2 is tissue-specific: mainly in testis but also in spleen, heart, kidney, pancreas, skeletal muscle, brain, liver, and leukocytes (Lin et al., 1998). High levels of expression are found in some types of cancer cells (Mathupala et al., 2004; Lee et al., 2012; Valença et al., 2015).

Partial localization of MCT1 and MCT2 in rat liver peroxisomes was initially reported by McClelland et al. (2003). This localization supported the hypothesis of a peroxisomal lactate shuttle involved in the peroxisomal exchange of lactate and pyruvate that would be required for the reoxidation of NADH, produced by peroxisomal beta-oxidation (see also “Shuttle Systems”). This hypothesis was supported by the stimulation of palmitoyl-CoA beta-oxidation of purified peroxisomes upon pyruvate supplementation. Also, an inhibitor of MCT proteins, α-cyano-4-hydroxycinnamate, was able to partly inhibit this stimulation (McClelland et al., 2003).

More recently, Valença et al. (2015) showed a peroxisomal localization of MCT2 in malignant prostate cells but not in immortalized benign prostate cells using immunofluorescence analysis. MCT1 was also studied in this study, but was mainly found in the nucleus and plasma membrane with only a minor localization in peroxisomes in prostate cancer cell lines. Involvement of MCT2 in cancerogenesis was indicated for prostate cancer (Pértega-Gomes et al., 2015), colorectal cancer (Lee et al., 2012), and glioblastoma (Mathupala et al., 2004), while MCT1 is a known biomarker for carcinogenesis and its overexpression is associated with breast, bone, colon, renal, and bladder cancers (Park et al., 2018). Apart from these two reports, no other information on a possible peroxisomal localization of MCT1 and MCT2 has appeared.

### SLC25 Transporters

**PMP34** encoded by the *SLC25A17* gene is the human ortholog of the *Saccharomyces cerevisiae* peroxisomal ATP transporter Ant1p (Palmieri et al., 2001; Visser et al., 2002; Agrimi et al., 2011), the PMP47 protein of the yeast *Candida boidinii* (Wylin et al., 1998) and the peroxisomal CoA transporter from Zebrafish (Kim et al., 2019). PMP34 was localized to mammalian peroxisomes using immunofluorescence microscopy analysis (Wylin et al., 1998) and proteomic analysis (Islinger et al., 2007; Wiese et al., 2007; Gronemeyer et al., 2013). *In vitro* reconstitution of the human protein followed by substrate exchange studies revealed that this protein is able to transport CoA, FAD, and, to a lesser extent, NAD^+^ (Agrimi et al., 2011). Earlier work had suggested that the protein may serve as an ATP transporter, similar as its ortholog in *S. cerevisiae* (Visser et al., 2002), but this was disputed by Agrimi et al. (2011) (see “Peroxisomal ATP Transport”). The different metabolic functions of peroxisomes require the import of CoA, FAD, NAD^+^, and ATP; PMP34 is so far the only transporter identified in human peroxisomes that shows substrate specificity toward these cofactors. Two orthologs of PMP34 in zebrafish (SLC25A17 and SLC25A17−like proteins) were shown to act as a CoA transporters (Kim et al., 2019).

Recently, van Veldhoven et al. (2020) reported the characterization of a mouse with a complete, whole-body knock-out of PMP34. The loss of PMP34 did not cause an obvious phenotype in mice with a Swiss Webster genetic background. Peroxisomal α- and β-oxidation rates in PMP34-deficient fibroblasts or liver slices of the mice were not or only modestly affected and the peroxisomal content of cofactors like CoA, ATP, NAD^+^, thiamine-pyrophosphate, and pyridoxal-phosphate, based on direct or indirect data, appeared normal. However, when the knock-out mice were challenged with dietary phytol administration, this led to significantly higher levels of phytanic acid and pristanic acid in the liver of the PMP34 knock-out mice when compared to wild-type mice. The authors therefore concluded that PMP34 is important for the degradation of phytanic/pristanic acid and/or export of their metabolites, but could not resolve the actual function of the protein.

### SLC27 Transporters

Acyl-CoA synthetases catalyze the esterification of fatty acids into their corresponding CoA esters, after which the fatty acyl-CoAs become substrate for different enzymes, including acyl-CoA oxidases that catalyze the first step in beta-oxidation. Furthermore, as discussed above, acyl-CoAs are also substrates for several transport proteins, including the ABCD proteins. In humans, two very-long-chain acyl-CoA synthetases have been partially localized to the peroxisomal membrane. These include acyl-CoA synthetase 5 - **ACSVL5** (FATP4, SLC27A4) (Jia et al., 2007a) and acyl-CoA synthetase 1 – **ACSVL1** (FATP2, SLC27A2) (Uchiyama et al., 1996; Steinberg et al., 1999). ACSVL5 was shown to have substrate specificity for very-long-chain fatty acids (Herrmann et al., 2001; Jia et al., 2007a) and ACSVL1 for long-chain, very-long-chain, branched-chain, and n-3 unsaturated (C18:3 and C22:6) fatty acids (Uchiyama et al., 1996; Steinberg et al., 1999; Melton et al., 2011); most of these fatty acids need to be oxidized inside peroxisomes. In addition to their enzymatic function in fatty acid esterification, ACSVL proteins have been suggested to also function as fatty acid transport proteins (FATP) and have thus been classified as SLC proteins. This suggestion was based on the finding of an increased uptake of fatty acids in cells upon overexpression of these proteins (Schaffer and Lodish, 1994; Hall et al., 2005). However, Jia et al. (2007b) showed that ACSVL5 (FATP4, SLC27A4) is driving fatty acid uptake but is not a transporter *per se*. Indeed, the authors postulated that ACSVL5 facilitates transport by trapping fatty acids in the form of their CoA esters thereby generating a large gradient across the peroxisomal membrane for free fatty acids (“Pulling Mechanism”). Other studies support this mechanism also for other acyl-CoA synthetases (Mashek and Coleman, 2006; Tong et al., 2006). More recently, Narita et al. (2016) presented findings that support the hypothesis that some of the human acyl-CoA synthetases possess transporter activity which is not related to the enzymatic activity. This followed from the observation that human ACSVL4 and some other acyl-CoA synthetases were able to transport long-chain bases of sphingolipids that lack a carboxyl group, the acceptor for CoA, and thus cannot be esterified (Narita et al., 2016). Also, Melton et al. (2011) showed that one of the isoforms of ACSVL1 lacks the ATP-binding domain and thus is unable to activate VLCFA, while it is still able to transport fatty acids when expressed in yeast and Hek293 cells.

The function of ACSVLs in peroxisomes remains controversial (reviewed by Watkins and Ellis, 2012) and they seem mainly involved in the activation of fatty acids rather than transport. However, the topographic orientation of ACSVLs in the peroxisomal membrane was never resolved definitively (Lageweg et al., 1991; Steinberg et al., 1999; Watkins, 2008; Watkins and Ellis, 2012), thus it remains the question whether the actual catalytic domain of these enzymes is facing the cytosol or the peroxisomal matrix.

Interestingly, both ACSVL1 and ACSVL5 have a non-canonical PTS1 signal (ACSVL1 has a C-terminal tripeptide LKL, and ACSVL5 – EKL). Also, the C-terminal peptide of ACSVL1 has more affinity for Pex5 than a C-terminal peptide from catalase (Ghosh and Berg, 2010). However, PTS1 signals normally target peroxisomal matrix proteins to peroxisomes and it has not been demonstrated that this signal can also target membrane proteins like ACSVL1 and ACSVL5.

### Uncharacterized Putative Transporters

PXMP4 (PMP24) and PMP52 (TMEM135) are two peroxisomal membrane proteins that based on sequence similarity have been assigned to the Tim17 family (Žárský and Doležal, 2016). This family includes proteins that mediate different functions in multiple cellular compartments including mitochondria, plastids and peroxisomes.

**PXMP4** is an evolutionary highly conserved protein among eukaryotes (Žárský and Doležal, 2016) which was first identified in rat peroxisomes (Reguenga et al., 1999) and later in human peroxisomes in different proteomic studies (Islinger et al., 2007; Wiese et al., 2007; Gronemeyer et al., 2013). The function of PXMP4 is unknown, but weak homology of PXMP4 with some bacterial permeases suggests that this protein may be involved in peroxisomal metabolite transport (Visser et al., 2007b). So far, only few reports on possible functions of PXMP4 have appeared.

The generation of natural killer T cells in a mouse model of type 1 diabetes and systemic lupus erythematosus was found to be regulated by two different genetic loci; one of them including the *PXMP4* gene. The function of PXMP4 in NKT cell formation has remained unclear (Fletcher et al., 2008), but may be related to the finding that peroxisomal synthesis of ether-phospholipids is required for the generation of semi-invariant natural killer T cells (Facciotti et al., 2012). This would suggest a role of PXMP4 in transport of glycerol 3-phosphate and/or dihydroxyacetone-3-phosphate better known as glycerone-3-phosphate required for ether-phospholipid biosynthesis. Silencing of PXMP4 expression was found during the tumorigenesis of prostate cancer cells (Zhang et al., 2010) and upregulation of PXMP4 expression in arteries is associated with obesity (Padilla et al., 2014).

**PMP52** (TMEM135) is a peroxisomal membrane protein predicted to have eight transmembrane domains (Žárský and Doležal, 2016). PMP52 has high homology to the mitochondrial protein Tim17, which is responsible for the insertion of mitochondrial membrane proteins. The peroxisomal localization of PMP52 in mammalian cells was demonstrated in several proteomic studies (Islinger et al., 2007; Wiese et al., 2007) and confirmed by overexpression of Myc-tagged PMP52 in CHO (Islinger et al., 2007), Huh7 and RPE1 (Maharjan et al., 2020) cell lines. In mouse fibroblast cells and monkey kidney fibroblast-like cells (Cos-7), PMP52 was localized to vesicular structures connected to mitochondria (Lee et al., 2016). Unfortunately, in this study only mitochondrial but no peroxisomal fluorescent markers were used, which renders it likely that the vesicular structures are actually peroxisomes and the reported mitochondrial localization might well be a misinterpretation of a peroxisomal-mitochondrial tether structure.

As for PXMP4, also the role of PMP52 has remained unclear and only few reports on possible functions have appeared. PMP52 has been proposed to play a role in peroxisomal turnover based on the finding that overexpression of PMP52 affects peroxisomal morphology and number (Islinger et al., 2007), but knockdown of PMP52 did not affect peroxisomal abundance (Maharjan et al., 2020). Instead, PMP52 depletion led to a decrease of lysosomal-peroxisomal contact sites and to accumulation of cholesterol in lysosomes (Maharjan et al., 2020). Previous studies already showed involvement of peroxisomes in cellular redistribution of cholesterol (Chu et al., 2015), and a role of PMP52 in this pathway may be related to peroxisomal turnover, insertion of membrane proteins, formation of contact sites or transport of metabolites (e.g., cholesterol).

In PMP52 knockdown mice a few peroxisomal matrix proteins – ACAA1 and SCP2 – were mislocalized to the cytosol which suggests a role of PMP52 in peroxisomal matrix protein import (Renquist et al., 2018). In the same study, an increased level of triglycerides was found in HepG2 cells with a knockdown of PMP52 (Renquist et al., 2018). Finally, in two different mouse models in which either very long-chain acyl-CoA dehydrogenase (VLCAD) or long-chain acyl-CoA dehydrogenase (LCAD) were disrupted, PMP52 levels were significantly elevated. Both LCAD and VLCAD are mitochondrial enzymes that catalyze the first step in mitochondrial long-chain fatty acid beta-oxidation. The increased PMP52 protein levels may relate to a regulatory mechanism required for mouse survival (Exil et al., 2010).

### Peroxisomal Cofactors

#### Peroxisomal FAD Transport

FAD is an essential co-factor for enzymes involved in peroxisomal fatty acid beta-oxidation as well as peroxisomal oxidation of other substrates, including D-amino acids and L-pipecolic acid. FAD may be covalently linked to the enzymes, as is the case for PIPOX (peroxisomal sarcosine oxidase, also known as peroxisomal L-pipecolic acid oxidase) (Mihalik et al., 1991), or non-covalently linked, as is the case for most other peroxisomal oxidases (Lienhart et al., 2013).

**FIGURE 3:**
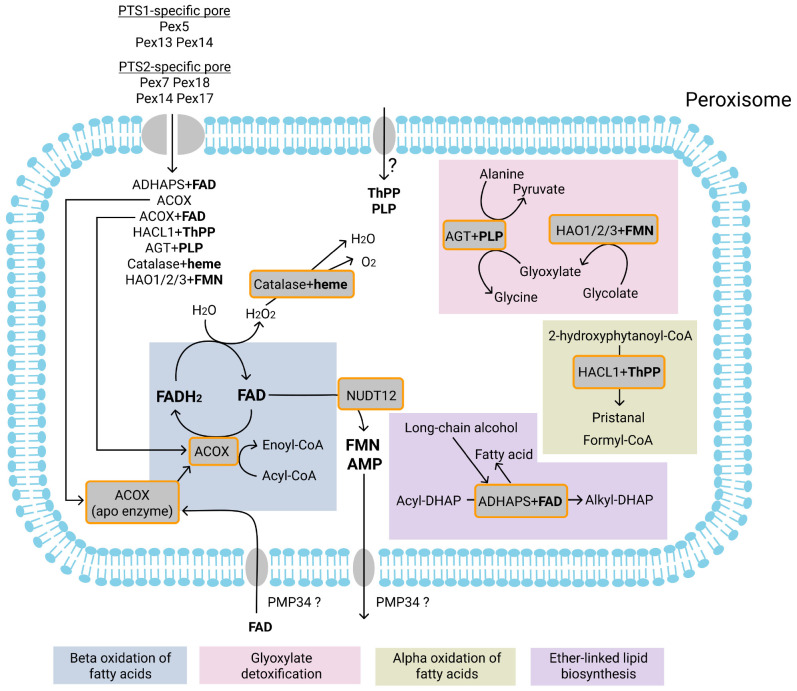
Currently known FAD, PLP, ThPP, FMN, and heme-dependent enzymatic reactions in the peroxisomes and transport of FAD, PLP, ThPP, heme, FMN and AMP. Most of the peroxisomal matrix proteins are imported into peroxisomes via a PTS1-specific pore. Proteins may be imported as monomers or in a fully folded form complexed with their cofactor. Cofactors that may be co-imported with proteins are FAD (with ACOX and ADHAPS proteins), FMN (HAO1, HAO2, HAO3), ThPP (with HACL1), PLP (with AGT), and heme (with catalase). It has also been suggested that the PMP34 transporter can import free FAD into peroxisomes. In the peroxisome, FAD forms an active enzyme with apoenzyme ACOX. The pyrophosphatase NUDT12 may mediate the degradation of FAD to FMN and AMP. FMN and AMP are subsequently exported from peroxisomes, probably by PMP34. Acyl-CoA oxidases (ACOX) mediate the first (dehydrogenation) reaction of the beta-oxidation. During dehydrogenation, FAD is reduced to FADH_2_. FADH_2_ is re-oxidized by direct transfer of electrons to O_2_, resulting in the production of H_2_O_2_. H_2_O_2_ is degraded by the heme-dependent enzyme catalase to H_2_O and O_2_. It is unclear whether free ThPP or PLP are imported into peroxisomes. Alkyl-dihydroxyacetonephosphate synthase (ADHAPS) catalyzes the exchange in acyl-dihydroxyacetonephosphate (acyl-DHAP) of the acyl chain with long-chain alcohol through a non-redox mechanism. The ThPP-dependent enzyme 2-hydroxyacyl-CoA lyase (HACL1) catalyzes the cleavage of 2-hydroxyphytanoyl-CoA into pristanal and formyl-CoA during peroxisomal alpha-oxidation of phytanoyl-CoA. The PLP-dependent enzyme alanine-glyoxylate aminotransferase (AGT) catalyzes the transamination of glyoxylate to glycine during glyoxylate detoxification. The FMN-dependent enzymes 2-hydroxyacid oxidases (HAO1, HAO2, HAO3) catalyze oxidation of glycolate to glyoxylate. Enzymatic reactions or molecules belonging to the same metabolic pathway are marked with background color and listed at the bottom.

In mitochondria, FAD import is mediated by an FAD transporter (Spaan et al., 2005). Two different mechanisms have been proposed for the import of FAD into peroxisomes; transport of FAD by PMP34 and co-import of FAD with the enzyme (Figure 3). The second mechanism was proposed based on the finding that peroxisomal matrix proteins may be imported into the peroxisomes as already fully folded proteins (Walton et al., 1995). One of the examples is acyl-CoA oxidase, which in the yeast *Yarrowia lipolytica* is imported into peroxisomes in the FAD-bound form as a heteropentameric complex (Titorenko et al., 2002). Acyl-CoA oxidases catalyze the first step in beta-oxidation, during which FADH2 is formed that subsequently is re-oxidized by direct transfer of electrons to O_2_, which results in the production of H_2_O_2_ within peroxisomes. H_2_O_2_ is subsequently converted to H_2_O and O_2_ by the peroxisomal enzyme catalase.

Ether-phospholipids are a class of phospholipids containing an ether bond at the *sn*-1 position of the glycerol backbone; the most abundant form of ether-phospholipids is plasmalogens (Wanders and Brites, 2010). A few peroxisomal enzymes are involved in the biosynthesis of ether-phospholipids. Of these, alkyl-dihydroxyacetonephosphate synthase (ADHAPS) catalyzes the exchange of the acyl chain with a long chain alcohol in acyl-dihydroxyacetonephosphate (acyl-DHAP). ADHAPS contains FAD as cofactor (de Vet et al., 2000) but during the enzyme reaction the redox state of FAD does not alter. Structural data showed that ADHAPS uses FAD to covalently trap substrates during the exchange reaction (Nenci et al., 2012). ADHAPS is targeted to peroxisomes via a PTS2 signal (Mizuno et al., 2013) and FAD may be co-imported with the protein.

#### Peroxisomal FMN Transport

Peroxisomes also contain the FMN-dependent enzymes HAO1, HAO2, and HAO3, which are 2-hydroxyacid oxidases (Jones et al., 2000; Recalcati et al., 2001; Murray et al., 2008). These enzymes are targeted to peroxisomes via a PTS1 signal. Similar as is the case for FAD, FMN is probably co-imported with the fully folded proteins into the peroxisomal lumen (Figure 3). Alternatively, FMN may be generated inside the peroxisomes by the FAD-degrading enzyme NUDT12 (see “Peroxisomal Solute Export”).

#### Peroxisomal ThPP Transport

The peroxisomal enzyme 2-hydroxyacyl-CoA lyase (HACL1) catalyzes the cleavage of 2-hydroxyphytanoyl-CoA into pristanal and formyl-CoA during peroxisomal alpha-oxidation of phytanoyl-CoA. HACL1 is a homotetrameric enzyme dependent on the cofactor thiamine pyrophosphate (ThPP) and Mg2+ (Foulon et al., 1999). HACL1 is targeted to peroxisomes by the PTS1 signal. Mutation analysis of HACL1 showed that HACL1 oligomerizes and is targeted to peroxisomes even when unable to bind ThPP (Fraccascia et al., 2011). Also, HACL1 activity can be increased when supplementing ThPP and MgCl2 during activity measurements (Foulon et al., 1999). Although it has not been elucidated whether HACL1 binds ThPP prior to or after import into peroxisomes, the above findings suggest that ThPP may be transported independently from HACL1. This is not mediated by PMP34, as this protein did not transport ThPP *in vitro* (Agrimi et al., 2011). In addition, the concentration of ThPP in peroxisomes isolated from the liver of PMP34-deficient mice was unchanged (van Veldhoven et al., 2020; Figure 3). In mitochondria ThPP is imported by a specialized ThPP transporter SLC25A19 (Lindhurst et al., 2006).

#### Peroxisomal PLP Transport

Peroxisomes in human liver contain the pyridoxal 5-phosphate (PLP)-dependent enzyme alanine-glyoxylate aminotransferase (AGT) encoded by the *AGXT* gene. AGT is targeted to peroxisomes by a non-canonical PTS1 signal. The crystal structure of the complex of Pex5 with AGT revealed that AGT in this complex contains PLP covalently attached to a Lys residue (Fodor et al., 2012), which suggests that covalently bound PLP is co-imported into peroxisomes with AGT (Figure 3).

Intracellular trafficking of PLP remains poorly understood, but it has been suggested that a selective mechanism of import of PLP into organelles, notably mitochondria, should also exist (Whittaker, 2016). Indirect evidence indicates that peroxisomal PLP levels are not affected in the liver of PMP34-deficient mouse, indicated that PMP34 is not involved in peroxisomal PLP import (van Veldhoven et al., 2020).

#### Peroxisomal Heme Transport

The prototypical peroxisomal protein catalase is a tetramer that contains 4 heme molecules. Heme is synthesized in the mitochondria. Free heme is probably cytotoxic and in the cytosol is sequestered by heme-binding proteins (Yuan et al., 2013). The majority of heme-dependent proteins bind heme in the cytosol. A few heme-dependent proteins are folded inside the ER lumen where they subsequently bind heme; it has been suggested that heme is transported into the ER through a mitochondria-associated endoplasmic reticulum membrane (MAM) (Asagami et al., 1994; Loth et al., 2020). So far there is no indication for free heme transport into peroxisomes, but there has been a few reports that suggest that catalase with complexed heme is assembled prior to peroxisomal import (Koepke et al., 2007; Otera and Fujiki, 2012; Okumoto et al., 2020) and thus imported via the peroxisomal oligomeric protein import route (Léon et al., 2006; Wolf et al., 2010).

#### Peroxisomal ATP Transport

**FIGURE 4:**
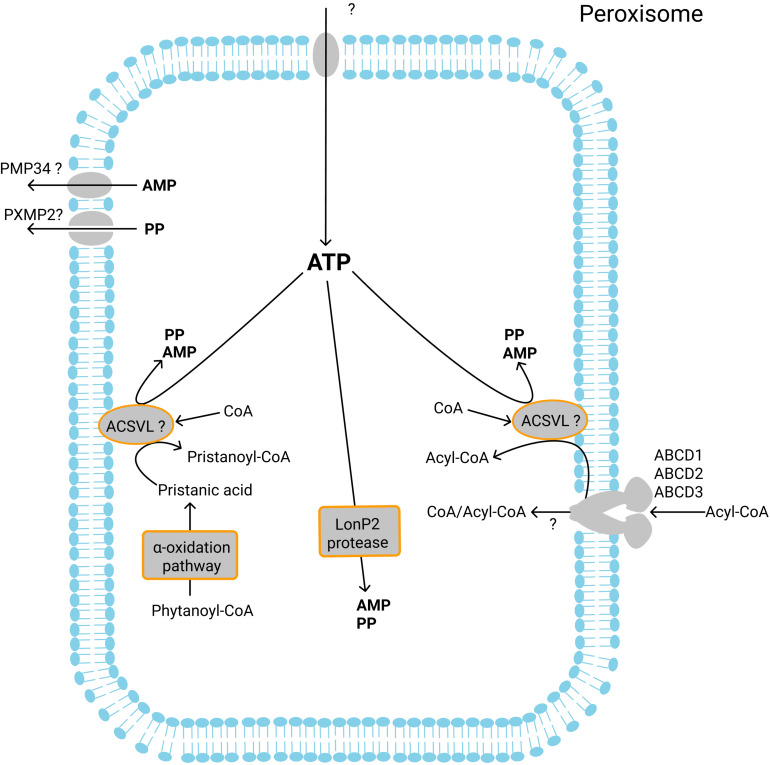
Currently known ATP-dependent enzymatic reactions in the peroxisomes and transport of ATP, AMP and PP. Intraperoxisomal ATP is required for the ACSVL-mediated activation of fatty acids after their import by the ABCD proteins. Pristanic acid formed during alpha-oxidation is activated inside peroxisomes to the corresponding CoA ester in an ATP-dependent reaction. Finally, the peroxisomal protease LonP2 hydrolyzes ATP. It is unknown which transporter mediates the import of ATP into peroxisomes. The products of ATP hydrolysis – AMP and PP are exported from peroxisomes most probably by PMP34 and PXMP2, respectively.

Several peroxisomal processes require the intraperoxisomal presence and hydrolysis of ATP (Figure 4). In yeast, peroxisomal import of long-chain fatty acids is mediated by the peroxisomal ABC half transporters Pxa1/Pxa2. However, medium-chain fatty acids are transported into peroxisomes as free fatty acids without involvement of Pxa1/Pxa2. To become substrate for beta-oxidation, the medium-chain fatty acids must first be activated into their corresponding CoA esters inside the peroxisome. This activation is catalyzed by peroxisomal acyl-CoA synthetase Faa2 (fatty acid activation protein 2) (Hettema et al., 1996) which catalyzes the ATP-driven synthesis of acyl-CoA esters and thus constitutes an intraperoxisomal ATP-consuming process. In humans, the fatty acids that are beta-oxidized in peroxisomes, including VLCFAs and long branched-chain fatty acids are converted into their corresponding CoA esters prior to import into the peroxisomes, which is mediated by the peroxisomal ABCD proteins.

Recent findings in yeast (van Roermund et al., 2012) and plants (Fulda et al., 2004; de Marcos Lousa et al., 2013) revealed that the ABCD-protein mediated transport mechanism may be more complicated than originally proposed and may require intraperoxisomal ATP. Similar as in humans, the activation of fatty acids to acyl-CoA is a prerequisite for ABC protein-mediated transport into plant peroxisomes. In plants, this import is mediated by the ABC transporter Comatose (also known as AtPxa1, Ped3, ACN2). Interestingly, Comatose was found to exhibit acyl-CoA thioesterase activity, which removes the CoA from the fatty acyl-CoA esters. The thioesterase activity seems to be required for the fatty acid transport as the loss of the activity due to targeted mutagenesis leads to the inability of Comatose to transport fatty acids (Carrier et al., 2019). Moreover, functional expression of Comatose in *S. cerevisiae* cells deficient for Pxa1/Pxa2 showed that the peroxisomal import of fatty acids is accompanied by the release of CoA and subsequently the reactivation of fatty acids to acyl-CoA, which is dependent on intraperoxisomal ATP (de Marcos Lousa et al., 2013; Carrier et al., 2019). Incubation of yeast cells with ^18^O-labeled H_2_O also showed that Pxa1/Pxa2-mediated import of fatty acids is accompanied by hydrolysis of the ester bond and subsesquent esterification (van Roermund et al., 2012).

A similar mechanism also seems to apply to human peroxisomal fatty acid import, as thioesterase activity was also reported for human peroxisomal ABCD proteins (Okamoto et al., 2018) and functional expression of human ABCD1 in *S. cerevisiae* cells deficient for Pxa1/Pxa2 showed that the peroxisomal import of C24:0 depends on the intraperoxisomal ATP-dependent acyl-CoA synthetase Faa2p (van Roermund et al., 2012). In contrast, Wiesinger et al. (2013) reported that the beta-oxidation of VLCFA-CoA in purified human peroxisomes was dependent on NAD^+^ but not on CoA, which may suggest that CoA is released in the peroxisomal matrix during the ABCD1-mediated transport of acyl-CoAs across the peroxisomal membrane. Taken together, whether hydrolysis of the CoA ester bond is required for ABCD transporter-mediated acyl-CoA transport and, if so, which intraperoxisomal Acyl-CoA synthetase is involved in the re-activation of fatty acids in human peroxisomes remains to be resolved in the future (see “SLC Family of Mitochondrial Solute Transporters”) (Figure 1A).

3-methyl-fatty acids cannot be metabolized by conventional beta-oxidation, but first need to undergo α-oxidation to become a substrate for beta-oxidation. The prototypical fatty acid that undergoes alpha-oxidation is the branched-chain fatty acid phytanic acid (3,7,11,15-tetramethylhexadecanoic acid), which is abundant in dairy products, ruminant fats, and certain fish. Phytanic acid is most likely imported into peroxisomes as CoA ester by ABCD3 (PMP70). Once inside peroxisomes, phytanoyl-CoA is converted to pristanoyl-CoA after one cycle of α-oxidation. During the final steps of the peroxisomal α-oxidation pathway, pristanic acid is activated to pristanoyl-CoA. This activation requires intraperoxisomal ATP and is most probably mediated by ACSVL1 (reviewed by Wanders et al., 2011). It should be noted that pristanic acid as derived from other sources than phytanic acid, can also can be activated outside the peroxisome (Wanders et al., 1992) and is subsequently transported as pristanoyl-CoA into peroxisomes by ABCD3 (PMP70) (Ferdinandusse et al., 2015; Figure 4).

In addition to its requirement for fatty acid oxidation, peroxisomal ATP is known to be required for at least one additional enzymatic reaction inside the peroxisomal lumen (Figure 4). LonP2 protease is an ATP-dependent enzyme that was found in peroxisomes of human, rat, mouse, *Caenorhabditis elegans*, *Penicillium chrysogenum*, and yeast (Kikuchi et al., 2004; Aksam et al., 2007; Bartoszewska et al., 2012) and has been shown to be responsible for the degradation of proteins that are damaged by oxidation (Bartoszewska et al., 2012) and also appears to be involved in sorting and processing of PTS1-containing proteins (Omi et al., 2008).

Because no ATP-forming enzymes are known to reside inside peroxisomes, the ATP required for intraperoxisomal ATP-consuming processes needs to be transported into the peroxisomes. In *S. cerevisiae*, the protein Ant1p was located in the peroxisomal membrane and shown to function as a peroxisomal adenine nucleotide transporter (Palmieri et al., 2001; van Roermund et al., 2001). Based on sequence similarity, the already mentioned SLC transporter PMP34 (SLC25A17) was identified as the human ortholog of yeast Ant1p, and subsequently shown to be located in mouse peroxisomes (Wylin et al., 1998). Functional expression showed that this PMP34 can partially rescue the ATP-dependent medium-chain fatty acid beta-oxidation in Ant1p-deficient mutants of *S. cerevisiae*. Although reconstitution of PMP34 in lipid vesicles showed the ability of PMP34 to transport ATP *in vitro*, albeit with low efficiency (Visser et al., 2002), a more recent detailed *in vitro* analysis of reconstituted PMP34 indicated that this protein is most probably a transporter of CoA, FAD, and, to a lesser extent, NAD^+^ but is less likely involved in ATP transport (Agrimi et al., 2011).

Peroxisomal transport of CoA, NAD(H), and NADP(H) are discussed in the chapters “SLC25 Transporters” and “Shuttle Systems.”

## Shuttle Systems

### Peroxisomal NAD^+^/NADH Shuttle

In peroxisomes, NADH is produced from NAD^+^ during the third step of beta-oxidation, involving the conversion of 3-hydroxyacyl-CoA to beta-ketoacyl-CoA, and during alpha-oxidation, involving the conversion of pristanal to pristanic acid. It remains unclear how NAD(H) is imported into peroxisomes. *In vitro* experiments indicated that net transport of NAD^+^ may be mediated by PMP34 (Agrimi et al., 2011), but expression of a PARP-based NAD^+^ biosensor targeted to peroxisomes in PMP34-deficient mouse fibroblasts showed that NAD^+^ content is not reduced (van Veldhoven et al., 2020). In plants, the peroxisomal transporter PXN imports NAD^+^ (Bernhardt et al., 2012), which suggests that a specific NAD^+^ transporter may also be present in human and yeast peroxisomes. However, although there are no examples for this, we cannot exclude that NAD(H) can be co-imported into peroxisomes with proteins, similar as has been described for FAD co-import (Figure 2). To maintain intraperoxisomal NAD(H) homeostasis, the generated NADH needs to be reoxidized into NAD^+^. This is most probably accomplished by an NAD^+^/NADH shuttle system similar as has been described for mitochondria. Although already suggested in Gee et al. (1974), this has not yet been conclusively demonstrated for mammalian peroxisomes in contrast to yeast, in which the existence of such a NAD(H)-redox shuttle was shown 25 years ago (van Roermund et al., 1995). Also for plants, the involvement of a peroxisomal NAD^+^/NADH shuttle was shown (Pracharoenwattana et al., 2007, 2010).

The requirements of an NAD^+^-shuttle system are the presence of (1) an NAD^+^-dependent dehydrogenase at either side of the membrane catalyzing the same reaction but in opposite directions, (2) metabolites that are substrate or products of the dehydrogenases and which can be transported across the peroxisomal membrane and (3) a transport system which may involve multiple carrier proteins that mediates exchange of the dehydrogenase substrate(s) and product(s).

As to requirement 1, several NAD^+^-dependent dehydrogenases that could be involved in NADH reoxidation, have been reported to reside in human peroxisomes, including lactate dehydrogenase B, malate dehydrogenase 1 and glycerol-3-phosphate dehydrogenase 1 (Figure 2). Each of these enzymes will be discussed below. As to requirement 2, several low-molecular-weight compounds (metabolites) that can play a role in such a shuttle system are probably located in human peroxisomes: malate, oxaloacetate, glycerol 3-phosphate, dihydroxyacetone phosphate, alcohol, lactate, pyruvate, serine, glycerate, glycerol, isocitrate. As to requirement 3, so far no specific transporter systems have been demonstrated in human peroxisomes that could mediate the exchange of the substrates of the dehydrogenases. However, as most of these metabolites have low molecular weights, they probably can diffuse via the size-selective channel-forming proteins discussed above (Antonenkov and Hiltunen, 2012; Figure 2).

### Lactate Dehydrogenase (LDH)

The human genome contains three different genes coding for LDH isoenzymes, LDHA, LDHB, and LDHC respectively. They most probably originate from gene duplications of a common ancestral gene during evolution. LDH catalyzes the NAD(H)-dependent interconversion of lactate and pyruvate. The functional LDH enzyme complex is a tetramer composed of different combinations of LDHA and LDHB subunits (LDHA4, ADHA3B1, LDHA2B2, LDHA1B3, LDHB4). LDHA is particularly abundant in skeletal muscle and liver while LDHB is the primary form in cardiac muscle. A third isoenzyme, LDHC, is expressed in testis only. LDHA and LDHB are predominantly cytosolic enzymes, but there are some observations suggesting a mitochondrial and nuclear localization (Maki et al., 2000; Passarella et al., 2014; Liu et al., 2018).

The first reports on a peroxisomal localization of LDH, including enzyme activity measurements in isolated peroxisomes, were already in the 1970s (McGroarty et al., 1974). The first indication that LDH may be involved in peroxisomal NADH reoxidation came from the observation that pyruvate causes a stimulatory effect on the peroxisomal beta-oxidation of erucoyl-CoA (C22:1-CoA) in intact rat peroxisomes (Osmundsen, 1982a, b). Later it was shown that addition of pyruvate to the reaction medium of purified peroxisomes led to complete reoxidation of NADH. This reoxidation could be inhibited by the LDH-inhibitor oxamate, suggesting the involvement of a peroxisomal LDH in the NADH reoxidation (Baumgart et al., 1996). The same group also reported the peroxisomal presence of an LDH4A isoform using isoelectric focusing and immunogold labeling (Baumgart et al., 1996; Fahimi et al., 1996). Finally, proteomic studies of human and rat peroxisomes demonstrated the presence of LDHA in peroxisomes (Wiese et al., 2007; Gronemeyer et al., 2013). This peroxisomal localization was supported by the finding of LDH activity in the purified peroxisomal fractions and a clear colocalization of over-expressed LDHA C-terminally fused to DsRed with a peroxisomal reporter protein EGFP-SKL (Gronemeyer et al., 2013).

Because LDHA and LDHB both lack prototypical peroxisomal targeting sequences required for the import of peroxisomal matrix proteins, the mechanism of import of LDH into the peroxisome has long remained unclear. However, translational read-through of the stop codon was found to result in an alternative isoform of LDHB with a C-terminal extension of six amino acids including a strong PTS1 signal that can target this isoform to the peroxisome (Schueren et al., 2014). Because LDHA and LDHB form tetrameric complexes (Gladden, 2004), it may be well possible that LDHA is co-transported with LDHB into peroxisomes by means of a piggy-back mechanism, which has previously been described as a peroxisomal import mechanism for several other peroxisomal proteins lacking a peroxisomal targeting sequence (review by Thoms, 2015).

### Malate Dehydrogenase (MDH)

The main function of MDH is the reversible catalysis of malate to oxaloacetate using NAD^+^/NADH as cofactor. The human genome contains two different MDH genes: *MDH1* and *MDH2*. Mitochondrial MDH2 functions as part of the Krebs cycle and the cytoplasmic MDH1 supports the malate–aspartate shuttle across the mitochondrial inner membrane.

In contrast to humans, *S. cerevisiae* has three genes encoding MDH enzymes: *Mdh1* codes for the mitochondrial MDH enzyme, *Mdh2* codes for the cytoplasmic enzyme, and *Mdh3* encodes the peroxisomal form. NADH reoxidation in peroxisomes of *S. cerevisiae* is achieved through a malate/oxaloacetate shuttle system involving peroxisomal Mdh3 and cytosolic Mdh2 (van Roermund et al., 1995). In the plant peroxisomes, the malate/oxaloacetate shuttle is also essential for reoxidation of NADH (Pracharoenwattana et al., 2007, 2010).

Proteomic studies of human and rat peroxisomes revealed that MDH1 is also localized in peroxisomes (Wiese et al., 2007; Gronemeyer et al., 2013). Similar as for LDHB (see above), MDH1 does not poses a prototypical peroxisomal targeting signal, but the protein can be extended by translational read-through of the stop codon generating a C-terminal PTS1 sequence which then targets this isoform to peroxisomes (Lingner et al., 2016). Thus, in analogy to yeast and plants, human peroxisomes may possess a malate-oxaloacetate shuttle system for the reoxidation of NADH, but this has yet not been demonstrated.

### Glycerol-3-Phosphate Dehydrogenase (GPD)

In addition to a malate/oxaloacetate shuttle, a Gpd1 (Glycerol-3-phosphate dehydrogenase 1)-based shuttle for peroxisomal NADH reoxidation was described for yeast (Al-Saryi et al., 2017). Gpd1 is targeted to the peroxisome via a PTS2 signal (Effelsberg et al., 2015; Kumar et al., 2016). This Gpd1p-based shuttle relies on the transport of glycerol 3-phosphate and dihydroxyacetone phosphate. The contribution of the two different NAD(H)-redox systems to the overall re-oxidation of intraperoxisomal NADH was found to depend on the growth medium (Al-Saryi et al., 2017). It is not yet clear why yeast would need two shuttle systems in peroxisomes, but the functioning of the shuttle systems may depend on the actual availability of substrates involved (Valadi et al., 2004). Important to add in this respect is that the Gpd1-based shuttle appears to be constitutive whereas the Mdh-based shuttle is inducible, notably when the yeast are cultured on oleate, the degradation of which relies on peroxisomal beta-oxidation.

In humans, there are two GPD genes known, GPD1 and GPD2. GPD1 catalyzes the reversible conversion of dihydroxyacetone phosphate (DHAP) to glycerol-3-phosphate (G3P) and uses NADH/NAD^+^ as cofactor. GPD1 forms the G3P-dependent mitochondrial shuttle, which transfers reducing equivalents from the cytosol into mitochondria where G3P is converted back into DHAP by an intramitochondrial membrane-bounded GPD2, which, in contrast to GPD1, reduces flavin adenine dinucleotide (FAD) to FADH_2_. This mitochondrial shuttle is taking place mainly in the brain, brown adipose tissue, and skeletal muscle of mammals (Mráček et al., 2013).

GPD1 was first suggested to be involved in the reoxidation of NADH in mammalian peroxisomes in 1974, based on the finding of its activity in peroxisomes isolated from rat, chicken or dog livers and rat kidney (Gee et al., 1974; Antonenkov, 1989). In two of the proteomic studies done for mammalian peroxisomes, Gpd1 was found in peroxisomes (Wiese et al., 2007; Gronemeyer et al., 2013), suggesting that Gpd1 may have been responsible for the enzymatic activity found by Gee et al. (1974). It is unclear how Gpd1 is transported into peroxisomes; it does not possess a prototypical peroxisomal targeting sequence and also does not create one by means of translational read-through.

In summary, at least three different dehydrogenases may form a peroxisomal NADH/NAD^+^ shuttle – MDH1, LDHB, and GPD1. However, the actual involvement of any of the peroxisomal enzymes in NADH homeostasis still remains to be shown experimentally.

### Aspartate Aminotransferase

A peroxisomal localization of aspartate aminotransferase was shown in yeast (Verleur et al., 1997) and in *Drosophila melanogaster* (Baron et al., 2016), suggesting that, similar to mitochondria, peroxisomes may contain a malate-aspartate shuttle. However, deletion of the gene encoding aspartate aminotransferase in *S. cerevisiae* did not cause a beta-oxidation defect in contrast to the deletion of Mdh3 (Verleur et al., 1997). Aspartate aminotransferase is probably not localized in human peroxisomes, rendering it unlikely that a malate-aspartate shuttle is involved in human peroxisomal NAD^+^/NADH homeostasis.

### Peroxisomal NADP^+^/NADPH Shuttle

In addition to saturated fatty acids, peroxisomes also play a role in the beta-oxidation of mono-/polyunsaturated fatty acids (review by van Veldhoven, 2010). To allow the beta-oxidation of unsaturated fatty acids to proceed, the double bound(s) of these fatty acids are reduced by the peroxisomal enzyme 2,4-dienoyl-CoA reductase (DECR2). This reduction requires the oxidation of NADPH into NADP^+^.

Another peroxisomal NADPH-dependent enzyme is *trans-*2-enoyl-CoA reductase (TER), encoded by the *PECR* gene. TER converts phytenoyl-CoA into phytanoyl-CoA which then becomes a substrate for the peroxisomal alpha-oxidation system (Gloerich et al., 2006).

It is unknown how NADP(H) is imported into the peroxisomes. NADP(H) may be imported into peroxisomes by the specific yet not identified transporter. Still, co-import of NADP(H) with NADP(H)-dependent proteins cannot be excluded as well (Figure 2). For the reduction of NADP^+^ back to NADPH, a peroxisomal 2-oxoglutarate/isocitrate NADP(H)-redox shuttle has been proposed (Figure 2).

In yeast, the peroxisomal 2-oxoglutarate/isocitrate NADP(H)-redox shuttle consists of a cytosolic and a peroxisomal isocitrate dehydrogenase, the latter of which is essential for beta-oxidation of unsaturated fatty acids (van Roermund et al., 1998). Isocitrate dehydrogenase 1 (IDH1) is also found in peroxisomes of rat (Yoshihara et al., 2001), mouse (Wiese et al., 2007), and humans (Geisbrecht and Gould, 1999; Gronemeyer et al., 2013), which suggests that also mammalian peroxisomes contain a 2-oxoglutarate/isocitrate NADP(H)-redox shuttle, similar as in yeast. Isocitrate dehydrogenase 1 has a double localization in the cytosol and peroxisomes in human liver cells and may form an NADP(H)-redox shuttle (Geisbrecht and Gould, 1999). Functional reconstitution of bovine kidney peroxisomal membrane proteins in proteoliposomes showed that peroxisomes are permeable for 2-oxoglutarate and isocitrate (Visser et al., 2006), which is in favor of a peroxisomal 2-oxoglutarate/isocitrate NADP(H)-redox shuttle. Transport of these solutes is probably mediated by one of the peroxisomal size-selective pore-forming proteins discussed above.

## Peroxisomal Solute Export

In addition to the import, peroxisomes also need to export metabolites and cofactors.

In human peroxisomes, beta-oxidation of VLCFAs leads to the formation of medium-chain fatty acyl-CoAs, acetyl-CoA, and propionyl-CoA, which for further metabolism in mitochondria need to be exported from peroxisomes. The export of acyl-CoAs may occur as free fatty/monocarboxylic acids following hydrolysis of the CoA esters and/or as carnitine esters, following exchange of carnitine for CoA.

The hydrolysis of acyl-CoA esters to free fatty acids in peroxisome is mediated by thioesterases (Figure 1A). In humans, two thioesterases have been found in peroxisome – ACOT4 and ACOT8. ACOT8 preferentially reacts with medium and long-chain fatty acyl-CoAs, whereas ACOT4 hydrolyzes succinyl-CoA, glutaryl-CoA, and long-chain fatty acyl-CoAs. Although it has been hypothesized that free fatty acids formed after hydrolysis by thioesterases can leave the peroxisome (Hunt et al., 2012), it is not clear if this is by simple diffusion or by a specific transport mechanism. Also, acyl-CoA synthetases localized on the cytosolic side of the peroxisomal membrane may be involved in the export of fatty acids (see “SLC27 Transporters”). More recently, it was hypothesized that membrane contact sites between peroxisomes and mitochondria may be involved (Schrader et al., 2015). Monocarboxylic acids resulting from hydrolysis by the thioesterases, such as acetate and propionate, may be exported via one of the peroxisomal pore-forming proteins.

Apart from hydrolysis, acyl-CoAs can also be converted to their corresponding carnitine esters by carnitine acetyl - and carnitine octanoyl transferases (CRAT and CROT) (Westin et al., 2008; Figure 1A). The carnitine required for this reaction may be imported into human peroxisomes by one of the Organic Cation Transporters. For rodents, the Octn3 (Organic Cation Transporter 3) (SLC22A21) carnitine transporter was reported in peroxisomes (Lamhonwah et al., 2003; Januszewicz et al., 2009; Figure 1A). Cross-reactivity with an antibody against mouse Octn3 was observed in human fibroblasts, but absent in PEX19-deficient fibroblasts (which completely lack peroxisomal membranes), which could suggest a peroxisomal localization of human OCTN3 (Lamhonwah et al., 2005). However, a peroxisomal localization of OCTN3 could not be confirmed by another research group (van Veldhoven et al., 2020).

The acyl-carnitines generated inside peroxisomes need to be transported from peroxisomes to mitochondria, where they undergo further degradation. In mitochondria, the carnitine acyl-carnitine carrier (CACT, SLC25A20) is mediating the import of acyl-carnitine esters. It is unknown, however, how acyl-carnitine esters are exported from human peroxisomes. In yeast, the transport of acetyl-CoA to mitochondria is facilitated by the peroxisome-mitochondria contact sites (Shai et al., 2016, 2018).

Alpha-oxidation of branched-chain fatty acids in peroxisome leads to the formation of formyl-CoA and subsequent activation of pristanic acid leads to formation of AMP and pyrophosphate. Formyl-CoA can undergo spontaneous hydrolysis to formic acid and CoA (Croes et al., 1997), after which formic acid can be degraded further via catalase or exported out of peroxisomes and degraded via the folate-dependent pathway (Tephly, 1991). The AMP produced as a consequence of the activation of pristanic acid is probably exported in exchange for CoA, NAD^+^ and/or FAD by the peroxisomal carrier PMP34 (SLC25A17) (Agrimi et al., 2011). The generated pyrophosphate should also leave the peroxisome and may be exported by one of the size-selective pore-forming proteins (Visser et al., 2005; Antonenkov and Hiltunen, 2012) or also by PMP34 (SLC25A17) (Agrimi et al., 2011).

The synthesis of bile acids from cholesterol consists of 17 reactions which occur in different subcellular compartments, including the cytosol, endoplasmic reticulum, mitochondria, and peroxisomes (Vaz and Ferdinandusse, 2017). During the final steps of the synthesis, bile acid intermediates are imported into peroxisome via ABCD3 (PMP70) and subjected to one cycle of beta-oxidation during which choloyl-CoA and deoxycholoyl-CoA are produced. Choloyl-CoA and deoxycholoyl-CoA are subsequently converted to their corresponding glycine or taurine conjugates by peroxisomal bile acid-CoA:amino acid N-acyltransferase (BAAT) (Hunt et al., 2012), which then are exported from the peroxisomes. Experiments with proteoliposomes prepared from purified mammalian peroxisomes showed that this export is ATP-independent, but the identity of the transporter involved has remained unknown (Visser et al., 2007a). Glycine and taurine can probably diffuse into peroxisomes via one of the pore-forming proteins (see “Pore-Forming Proteins”) (Figure 1A).

An important co-factor involved in peroxisomal lipid metabolism is Coenzyme A. CoA is released during hydrolysis of acyl-CoA esters by the thioesterases (see above) and may be used again inside peroxisomes for other enzymatic reactions, such as the activation of pristanic acid, or be degraded by one of the peroxisomal Nudix Hydrolases. Two CoA-degrading Nudix Hydrolases – NUDT7 and NUDT19 (also known as RP2p) - have been identified in human peroxisomes (Ofman et al., 2006), of which NUDT7 is also found in the cytosol (Carreras-Puigvert et al., 2017). NUDT19 may have a dual localization in mitochondria and peroxisomes, although Shumar et al. (2018) reported that at least in the human cell line HEK 293 this enzyme is exclusively peroxisomal. NUDT19 is mainly found in the kidney and NUDT7 in the liver (Shumar et al., 2018). Both NUDT19 and NUDT7 are CoA diphosphohydrolases that degrade CoA and acyl-CoAs to 3′,5′-ADP and 4′-(acyl)phosphopantetheine (Ofman et al., 2006; Shumar et al., 2018). How these degradation products are exported out of peroxisomes is not clear. The importance of NUDT7 in peroxisomal metabolism was shown in metabolomics analysis of mice that overexpress NUDT7 in the liver, which revealed a decrease in peroxisomal beta-oxidation and bile acid biosynthesis rates (Shumar et al., 2019). Nudt19-/- mice showed a 20% increase in CoA in the kidney (Shumar et al., 2018; Figure 1A).

In addition to NUDT7 and NUDT19, also NUDT12 was reported in human peroxisomes. In contrast to the other two, however, NUDT12 is not involved in CoA degradation but it is a pyrophosphatase that mediates the degradation of NADH (to NMNH and AMP) (Figure 2) and NADPH (to NMNH and 2’,5′-ADP) (Figure 2) but also shows moderate activity with FAD (to FMN and AMP) (Figure 3) and NAD^+^ (to NMN^+^ and AMP) *in vitro* (Abdelraheim et al., 2003; Carreras-Puigvert et al., 2017). It was suggested that the products of these reactions leave the peroxisome through the size-selective pore-forming proteins (Antonenkov and Hiltunen, 2012). Alternatively, FMN and AMP may be exported by PMP34 (Agrimi et al., 2011) (see “SLC Family of Mitochondrial Solute Transporters”) (Figures 2, 3).

## Metabolite Transport Between Organelles

In recent years, it has become clear that organelles extensively communicate with each other via close physical interactions, known as membrane contact sites. Also, for peroxisomes a number of proteins involved in the formation of membrane contact sites with other organelles have been identified (Schrader et al., 2015; Schrader, 2019). Some of these proteins are facilitating the transport of metabolites between peroxisomes and other organelles.

Acyl-CoA binding domain-containing protein 5 (ACBD5) is an abundant peroxisomal membrane protein that interacts with the ER proteins VAPA and VAPB (Costello et al., 2017) (Hua et al., 2017) to constitute membrane contact sites between peroxisomes and the ER. Similar as in X-linked adrenoleukodystrophy, patients with ACBD5 deficiency accumulate VLCFA (Ferdinandusse et al., 2017; Yagita et al., 2017; Herzog et al., 2018). Based on these findings it was suggested that the acyl-CoA binding domain of ACBD5 binds C26-CoA, and other VLCFA-CoAs, which are synthesized by fatty acid elongation at the ER (Sassa and Kihara, 2014), and presents it to peroxisomal ABCD1 (Ferdinandusse et al., 2017). ABCD1 would then subsequently transport C26-CoA into the peroxisome (see “Fatty Acid Import and the Role of the ABCD Transporters”). This mechanism is also supported by the finding that ACBD5 binds VLCFA-CoA *in vitro* (Yagita et al., 2017).

Knockdown of ACBD5 or VAPA and VAPB in cells resulted in a decreased plasmalogen and cholesterol levels suggesting that ACBD5-VAP tethers may also be involved in the trafficking of intermediates of plasmalogen synthesis (Hua et al., 2017).

Metabolomics analysis of ACBD5-deficient fibroblasts also revealed decreased levels of ether phospholipids, including plasmalogens (Herzog et al., 2018). Since the first steps of plasmalogen synthesis are peroxisomal followed by further synthesis in the ER, the membrane contact sites between peroxisomes and ER may provide an effective way to channel the transport of the plasmalogen intermediates between peroxisomes and ER.

There has been considerable debate as to whether peroxisomes are involved in isoprenoid/cholesterol biosynthesis. The suggestion about the role of peroxisomes in cholesterol biosynthesis was based mainly on the claim that several of the enzymes of the mevalonate pathway are located in peroxisomes. However, comprehensive studies later showed that these enzymes are definitely not present in peroxisomes (Hogenboom et al., 2003a, b, 2004). Moreover, none of these enzymes were identified after proteomic analyses of purified peroxisomes. Hence, a decrease in cholesterol levels caused by the depletion of ACBD5 or VAPA and VAPB may be an indirect effect and should be interpreted with caution.

Acyl-CoA binding domain-containing protein 4 (ACBD4) is another peroxisomal membrane protein which shares 58% sequence identity with ACBD5 and, like ACBD5, also shows interaction with the VAPA and VAPB proteins, suggesting a role in membrane contact sites. The function of ACBD4 is less clear than for ACBD5 (Costello et al., 2017).

The group of Bao-Liang Song reported that peroxisomal PI(4,5)P2 mediates the formation of membrane contact sites with lysosomes (through Syt7 protein) (Chu et al., 2015) and ER (through extended-synaptotagmins 1-3) (Xiao et al., 2019), and postulated that the PI(3,4)P2 levels in peroxisomes are regulated by phosphatidylinositol 5-phosphate 4-kinase type-2 alpha (PIP4K2A) (Hu et al., 2018). However, a peroxisomal localization of PIP4K2A was not shown directly and the experimental approaches used and certain conclusions drawn in these studies were critically debated (van Veldhoven et al., 2015).

Given that peroxisomes play an important role in lipid metabolism it is not a surprise that peroxisomes also form membrane contact sites with lipid droplets. Chang et al. (2019) showed that these membrane contacts are formed by the M1 isoform of Spastin protein through interaction with ABCD1. In addition to the ABCD1-interacting region, M1 Spastin has a hairpin motif that allows interaction with lipid droplets, a microtubule interacting and trafficking (MIT) domain, and an ATPase-Associated with diverse cellular Activities (AAA) domain (Chang et al., 2019). Pulse-chase studies with fluorescent analogs of fatty acids showed that a knockdown of Spastin decreases trafficking of fatty acids to peroxisomes and overexpression of M1 Spastin increases trafficking (Chang et al., 2019). Overexpressed M1 Spastin not only forms a tether between organelles but also recruits ESCRT-III machinery proteins – IST1 (increased sodium tolerance 1) and CHMP1B (charged multivesicular body protein 1B) via the MIT domain (Chang et al., 2019). It was suggested that IST1 and CHMP1B are polymerizing on lipid droplet surface into cone-like structures, to extract lipids from lipid droplets and present them to ABCD1 (Chang et al., 2019; Henne, 2019).

The metabolic cooperation between peroxisomes and mitochondria requires that many metabolites are transported between the two organelles, including acetyl-CoA, propionyl-CoA, medium-chain acyl-CoA, reducing equivalents from NADH, glycolate, glyoxylate, phospholipids, H_2_O_2_, ATP, NAD^+^, and others. While some of these may be transported via diffusion through the cytosol, their channeling via contact sites between the organelles will increase the efficiency and specificity. Yet, so far no bona fide contact sides between these two organelles have been reported in mammalian cells. A recent study shows that ACBD2/ECI2 protein might form the molecular tether between organelles in mouse Leydig cells (Fan et al., 2016). The authors suggested that the ACBD2/ECI2 protein complex facilitates tethering of peroxisomes with mitochondria by interacting simultaneously with both the mitochondrial (through N-terminal targeting signal) and peroxisomal (through C-terminal targeting signal) protein import machinery (Fan et al., 2016). To further verify this mechanism of tether formation additional studies are required (Islinger et al., 2020).

As an alternative, small vesicles may mediate the transport of metabolites between organelles. It was shown that mitochondria-derived (Neuspiel et al., 2008; Braschi et al., 2010; Sugiura et al., 2014) and ER-derived (Sugiura et al., 2017) vesicles are transported from these organelles to peroxisomes and that both are necessary for the formation of new peroxisomes in peroxisome-deficient human fibroblasts (Sugiura et al., 2017). In wild-type cells, mitochondria-derived vesicles can also fuse with mature peroxisomes (Neuspiel et al., 2008; Braschi et al., 2010). It is possible that specific metabolites/cofactors are transported from mitochondria or ER by these vesicles (Neuspiel et al., 2008).

## Discussion

In this review, we have discussed the current knowledge and the most important gaps in our understanding of human peroxisomal metabolite and cofactor transport and contradictory findings collected over the past two decades. It is evident that future studies are required to address important questions including: (1) what is the physiological role of the pore-forming proteins in peroxisomal functioning, (2) what is the involvement of peroxisomal acyl-CoA synthetases in the import and export of fatty acids, (3) how do the different shuttle systems function and what is their physiological role, (4) how are metabolites exchanged between organelles, (5) how are bulky metabolites (ATP, NADP^+^, carnitine-esters, and other) and H_2_O_2_ transported across the peroxisomal membrane.

Our current knowledge of peroxisomal metabolite transport is mainly based on the identification of human genetic metabolic disorders, mouse and yeast models, and *in vitro* experiments with isolated peroxisomes or candidate proteins. Although some peroxisomal transporter proteins have been identified and characterized, there are still big gaps in our understanding of peroxisomal metabolite and cofactor transport.

One major bottleneck in the studies of peroxisomal metabolite transport is the inability to measure intraperoxisomal metabolites in a direct way. The classical approach of measuring metabolites in isolated organelles is challenging for peroxisomes as they are notorious for their *in vitro* leakiness and are, therefore, permeable to most of the metabolites during isolation. Recently developed genetically encoded biosensors may become a new powerful tool to study peroxisomal transporters. A variety of available biosensors enable detection of ATP, NAD^+^/NADH, NADP^+^/NADPH, lactate, pyruvate, bile acids, glutathione, pH, H_2_O_2_, NO, and many other metabolites and cofactors *in vivo*. In comparison to measurements in cell lysates, biosensors can provide insight into the distribution of metabolites between different subcellular compartments without subcellular fractionation. They provide the possibility of (1) real-time observations of the dynamics of subcellular metabolism in individual cells or even individual peroxisomes; (2) simultaneous targeting of biosensors to different organelles to observe subcellular transport and organelles interaction; (3) simultaneous measurement of a few different metabolites inside peroxisome; (4) measurement of metabolites in different dynamic ranges with low/high-affinity biosensors; (5) high-throughput screenings using the FACS technique.

Recent advances in genome editing also provide novel opportunities for studying the role of peroxisomal membrane proteins using a classical bottom-up approach. As functional redundancy may be an important feature of peroxisomal transport it may be required to generate cell models with multiple gene deletions employing the CRISPR genomic editing to identify and elucidate the specific transport functions of candidate proteins.

## Author Contributions

SC, HW, LI, and CR conceived the format of the manuscript. SC wrote the draft of the manuscript. HW, LI, RW, and CR made significant contributions and revisions to the manuscript. SC and LI prepared the figures. All authors approved the submitted version.

## Conflict of Interest

The authors declare that the research was conducted in the absence of any commercial or financial relationships that could be construed as a potential conflict of interest.
